# Atypical Hemolytic Uremic Syndrome in the Setting of Acute Clostridium difficile Colitis

**DOI:** 10.7759/cureus.13244

**Published:** 2021-02-09

**Authors:** Anshu Wadehra, Samer Alkassis

**Affiliations:** 1 Internal Medicine, Wayne State University/Detroit Medical Center, Detroit, USA

**Keywords:** hus, clostridium difficile, atypical hemolytic uremic syndrome

## Abstract

Hemolytic uremic syndrome (HUS) is characterized by microangiopathic hemolytic anemia, thrombocytopenia, and acute renal failure. HUS can be secondary to multiple etiologies such as infections, medications, and immune processes. A rare, yet significant, etiology of HUS includes acute *Clostridium difficile* colitis. Here, we present a case of atypical HUS secondary to acute *C. difficile* colitis, successfully treated with hemodialysis and systemic corticosteroids. It is imperative that clinicians are cognizant of *C. difficile*-associated HUS given the overall rising incidence of acute *C. difficile* infections.

## Introduction

Hemolytic uremic syndrome (HUS), as defined by the triad of microangiopathic hemolytic anemia, thrombocytopenia, and acute kidney injury, has been shown to be the result of many bacterial infections [1]. Additionally, HUS can be secondary to viral infections, medications, and immune processes [2-4]. The most commonly reported causative bacteria include *Escherichia coli* O157:H7, *Shigella dysenteriae*, *Salmonella typhi*, and *Streptococcus pneumoniae* [3]. *Clostridium difficile* has been rarely reported as a causative microbe in the development of HUS in the adult population [3]. Treatment of this entity typically involves supportive care; however, hemodialysis and blood transfusions may be required [5]. We present a case of an adult patient who was diagnosed with HUS secondary to *C. difficile* colitis.

## Case presentation

The patient was a 65-year-old male with significant medical history of immunoglobulin G multiple myeloma and hypertension, who presented to the hospital with three days of generalized fatigue and multiple daily episodes of non-bloody, watery diarrhea. He had mild diffuse abdominal tenderness on physical examination. On admission, laboratory workup was significant for acute renal failure, with serum creatinine of 7.77 mg/dL and blood urea nitrogen of 66 mg/dL. His serum creatinine measured five days prior to admission was 1.00 mg/dL. The patient was also noted to be anuric.

Further laboratory studies showed lactate dehydrogenase being significantly elevated to 3,835 U/L. Haptoglobin was low and total bilirubin was 1.12 mg/dL, with fractionation revealing it to be primarily unconjugated. Alanine aminotransferase and aspartate aminotransferase were elevated to 70 IU/L and 220 IU/L, respectively. Stool studies revealed a positive *C. difficile* polymerase chain reaction. Shiga toxin was negative. Stool culture was negative for *Salmonella *and *Shigella* as well.

**Figure 1:**
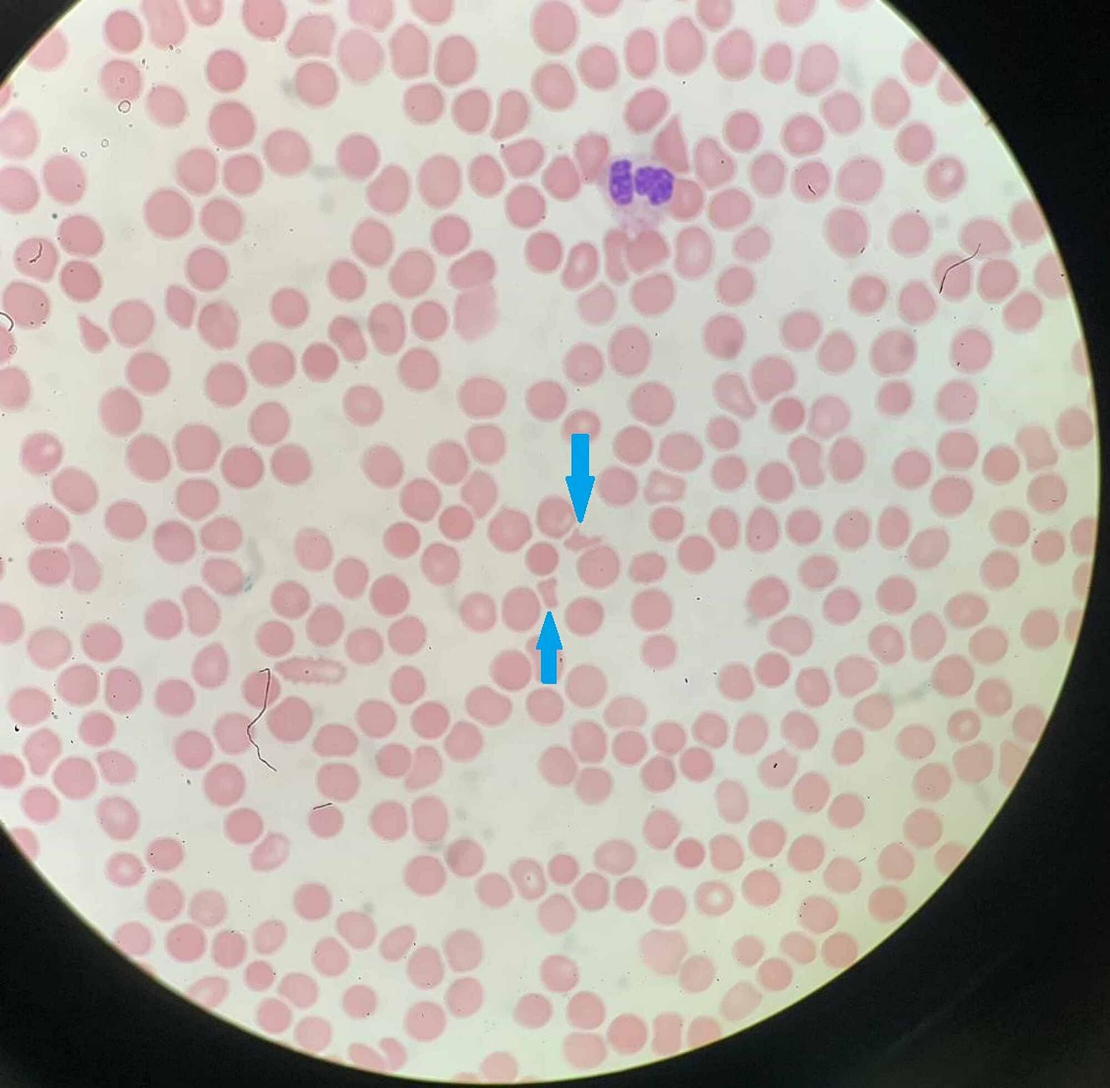
Peripheral blood smear revealing schistocytes (blue arrows).

Complete blood count revealed severe thrombocytopenia at 4,000/mm^3^. Platelet count was 274,000/mm^3^ five days prior to admission. Hemoglobin was reduced to 9.3 g/dL from his previous baseline of 12.0 g/dL, without any overt signs of bleeding. Mean corpuscular volume was 88.8 fL and INR was 1.00. ADAMTS13 activity was within normal limits at 93%. Coombs test was negative as well. Peripheral blood smear was reviewed and showed schistocytes (Figure 1). Based on these pertinent findings, a diagnosis of HUS was established.

During his hospital course, platelet count improved to 51,000/mm^3^ with supportive care, which later dropped to 19,000/mm^3^. Given his further drop in platelets, the decision was made to start the patient on high-dose intravenous (IV) methylprednisolone. The patient also required multiple red blood cell transfusions throughout his hospitalization to maintain his hemoglobin above a value of 7 g/dL.

The patient was treated for *C. difficile* infection with oral vancomycin. His renal function continued to decline during his hospital course, eventually requiring hemodialysis. He received five days of high dose IV methylprednisolone with resultant improvement of his platelet count to 86,000/mm^3^ on the day of discharge. Overall, his symptoms improved, and the patient was discharged with a prolonged steroid taper and continued outpatient hemodialysis.

## Discussion

HUS is a form of thrombotic microangiopathy characterized by microangiopathic hemolytic anemia, thrombocytopenia, and acute renal failure [6]. It was formerly classified into typical HUS, which is caused by Shiga toxin-producing *E. coli* and considered the most common type in children, and atypical HUS, or non-Shiga toxin HUS, which accounts for most of the adult cases [7]. The new classification was developed based on the pathophysiology and precipitating factors [8]; hereditary causes include complement gene mutations and cobalamin C deficiency. Acquired causes involve autoantibodies to complement factors, drug-induced, and infection.

Many infections have been associated with HUS including human immunodeficiency virus and *S. pneumoniae* [9,10]. *C. difficile* has been recognized as a rare trigger for HUS [3,11-13]. In contrast to *E-coli*-associated HUS, adults with *C. difficile*-associated HUS may have better prognosis than children [3]. The pathophysiology of *C. difficile*-associated HUS is not well understood; it was speculated that binding of cytotoxin A and B to specific receptors on colonic cell membranes induces apoptosis leading to mucosal breach and cytotoxin access into circulation, which may directly damage the renal microvasculature [3].

The diagnosis of HUS is clinically based on the presence of the classical triad, which is established by laboratory workup that includes a complete blood count with peripheral blood smear, renal functions studies, and urinalysis. Microangiopathic hemolytic anemia is defined by a hemoglobin level less than 10 g/dL with a negative Coombs test and a peripheral blood smear demonstrating schistocytes (up to 10% of red cells) and helmet cells [1]. Thrombocytopenia is characterized by a platelet count below 140,000/mm^3^. The degree of thrombocytopenia is unrelated to the severity of renal dysfunction. The severity of acute kidney injury ranges from hematuria and proteinuria to severe renal failure and oliguria.

The cornerstone management in HUS is supportive therapy, including red blood cell and platelet transfusion when clinically indicated, fluid and electrolyte management to maintain adequate intravascular volume and correct electrolyte abnormalities, and discontinuation of nephrotoxic medications [5]. Initiation of dialysis is indicated in patients with symptomatic uremia, severe fluid overload, or electrolyte abnormality that is refractory to medical therapy. Different conventional treatments have been used in *C. difficile*-associated HUS, including antibiotics, corticosteroids, and plasmapheresis with convenient response [14]. Eculizumab, a terminal complement inhibitor, has been shown to be effective and safe in atypical HUS, particularly in regards to long-term renal function and thrombotic microangiopathic events, based on a recent large prospective, observational, multicenter study [15]. Our patient completed 14-day course of oral vancomycin. In addition, he also received high-dose corticosteroids and was discharged on a tapering dose of prednisone.

## Conclusions

*C. difficile* infection may cause renal failure due to thrombotic microangiopathy which should be considered in the differential diagnosis of diarrhea-associated HUS. Treatment of this entity includes primarily supportive care; however, if inadequate response is observed, systemic corticosteroids may prove to be beneficial. With the rising incidence of pseudomembranous colitis, clinicians should be aware of the unusual manifestations of this common illness.
